# Central venous to arterial carbon dioxide gap as an indicator of oxygen debt in isovolemic anemia

**DOI:** 10.1186/cc9846

**Published:** 2011-03-11

**Authors:** S Kocsi, G Demeter, D Erces, J Kaszaki, Z Molnar

**Affiliations:** 1 University of Szeged, Hungary

## Introduction

Anemia can cause an imbalance in oxygen delivery (DO_2_) and consumption (VO_2_), which may be difficult to detect. Recently the venous to arterial carbon dioxide difference has been shown to be increased (>5 mmHg) in certain critically ill conditions [1,2]. No study has yet investigated its significance in severe normovolemic anemia. Therefore, the aim of this study was to investigate the course of the central venous to arterial carbon dioxide gap (dcvCO_2_) in isovolemic anemia.

## Methods

An experimental animal study on anesthetized Vietnamese mini-pigs. After splenectomy, mini-pigs (*n *= 13, weight range: 18 to 30 kg) were bled in five stages (~10% of estimated blood volume/5 minutes, T_0 _to T_5_) and blood loss was replaced by the same volume of colloid, after which hemodynamic measurements and blood gas analysis were performed.

## Results

The fall of hemoglobin was significant from the first bleeding, from T_0 _to T_5_: median = 125 (interquartile range = 113 to 134) to 49 (43 to 55) g/l, *P *< 0.05, respectively. Despite a significant increase in cardiac index by T_1 _(T_0 _= 2.6 (2.3 to 2.8) vs. T_1 _= 3.3 (2.7 to 3.6) l/minute/m^2^, *P *< 0.05), which remained so for the rest of the experiment, the O_2 _extraction (VO_2_/DO_2_) increased significantly only from T_3 _(T_0 _= 29 (18 to 33) vs. T_3 _= 35 (21 to 40)%, *P *< 0.05). Anemia was accompanied by a significant increase in dcvCO_2 _from T_0 _= 5 (3 to 9) to T_5 _= 6 (6 to 11) mmHg, *P *< 0.05. There was a strong significant correlation between VO_2_/DO_2 _and dcvCO_2_: *r *= 0.65, *r*^2 ^= 0.43, *P *< 0.001. Furthermore, dcvCO_2 _with a cut-off value >5 mmHg had a sensitivity of 69% and specificity of 82% to show a VO_2_/DO_2 _> 30%, and receiver operating characteristics showed an area under the curve of 0.787 ± 0.054 (CI: 0.682 to 0.892), *P *< 0.001, for the same VO_2_/DO_2 _threshold.

## Conclusions

To our best knowledge, this is the first study to show that dcvCO_2 _could be used to detect oxygen debt in isovolemic anemia.
